# Hippocampal Gene Expression Meta-Analysis Identifies Aging and Age-Associated Spatial Learning Impairment (ASLI) Genes and Pathways

**DOI:** 10.1371/journal.pone.0069768

**Published:** 2013-07-18

**Authors:** Raihan K. Uddin, Shiva M. Singh

**Affiliations:** Molecular Genetics Unit, Department of Biology, University of Western Ontario, London, Ontario, Canada; University College London, United Kingdom

## Abstract

A number of gene expression microarray studies have been carried out in the past, which studied aging and age-associated spatial learning impairment (ASLI) in the hippocampus in animal models, with varying results. Data from such studies were never integrated to identify the most significant ASLI genes and to understand their effect. In this study we integrated these data involving rats using meta-analysis. Our results show that proper removal of batch effects from microarray data generated from different laboratories is necessary before integrating them for meta-analysis. Our meta-analysis has identified a number of significant differentially expressed genes across age or across ASLI. These genes affect many key functions in the aged compared to the young rats, which include viability of neurons, cell-to-cell signalling and interaction, migration of cells, neuronal growth, and synaptic plasticity. These functional changes due to the altered gene expression may manifest into various neurodegenerative diseases and disorders, some of which leading into syndromic memory impairments. While other aging related molecular changes can result into altered synaptic plasticity simply causing normal aging related non-syndromic learning or spatial learning impairments such as ASLI.

## Introduction

Aging and age-associated cognitive impairments are complex and multifactorial and involve both genetic as well as environmental determinants. Both in humans and in animal models the process of normal aging often results in cognitive decline, with or without the presence of any aging related neurological disorders. Disorders related to cognitive impairments range from non-syndromic benign senescent forgetfulness to the syndromic memory loss that characterizes Alzheimer’s disease [1], [2], [3]. These manifestations are highly heterogeneous and individual, family, and population specific. They continue to increase with the current trend in longevity in most populations [4], [5], [6]. As such they are emerging as a major societal challenge. Attempts in the last decade to gain insight into aging and age-associated learning impairments have been aided by advances in genome-wide methods and technologies, particularly gene expression involving microarrays. Further, the hippocampus in the brain is integral to memory function including spatial memory both in humans and in rodents [7], [8]. It is greatly affected by aging, and is among the first to be affected during dementia [9], [10], [11], [12]. The microarray technology has been used widely, more specifically, to understand the gene expression changes related to aging and age-associated memory impairments in the hippocampus in humans [13] using post-mortem tissues [14] and in animal models such as rodents after behavioural training [4], [9], [15]. Results show that learning induces a complex reprogramming of gene expression, which is also affected by the aging processes. Moreover, the results of the individual studies are heterogeneous and often difficult to interpret. They often highlight different gene sets and pathways, have limited conclusions, and do not consider their broader implications that may go beyond individual experiments. It is therefore desirable to integrate results from these studies towards a consensus view of the genes affected and the molecular mechanisms underlying brain aging and age-associated learning impairments. This is now possible because of the availability of considerable amount of original microarray data in the public microarray data repositories, as well as the availability of improved statistical analytical methods. This study focuses on age-associated spatial learning impairment (ASLI). It uses original results from all available microarray gene expression data involving ASLI in rats using an inverse-variance meta-analysis approach [16]. The results establish that a large number of genes are differentially expressed across age and across spatial learning impairment. More importantly, they allow identification of pertinent lists of aging and ASLI related genes. Further, the follow up analysis has offered a novel insight into the underlying molecular pathways associated with aging and age-related non-syndromic memory impairments such as ASLI.

## Methods

### Data Selection

**Figure 1:**
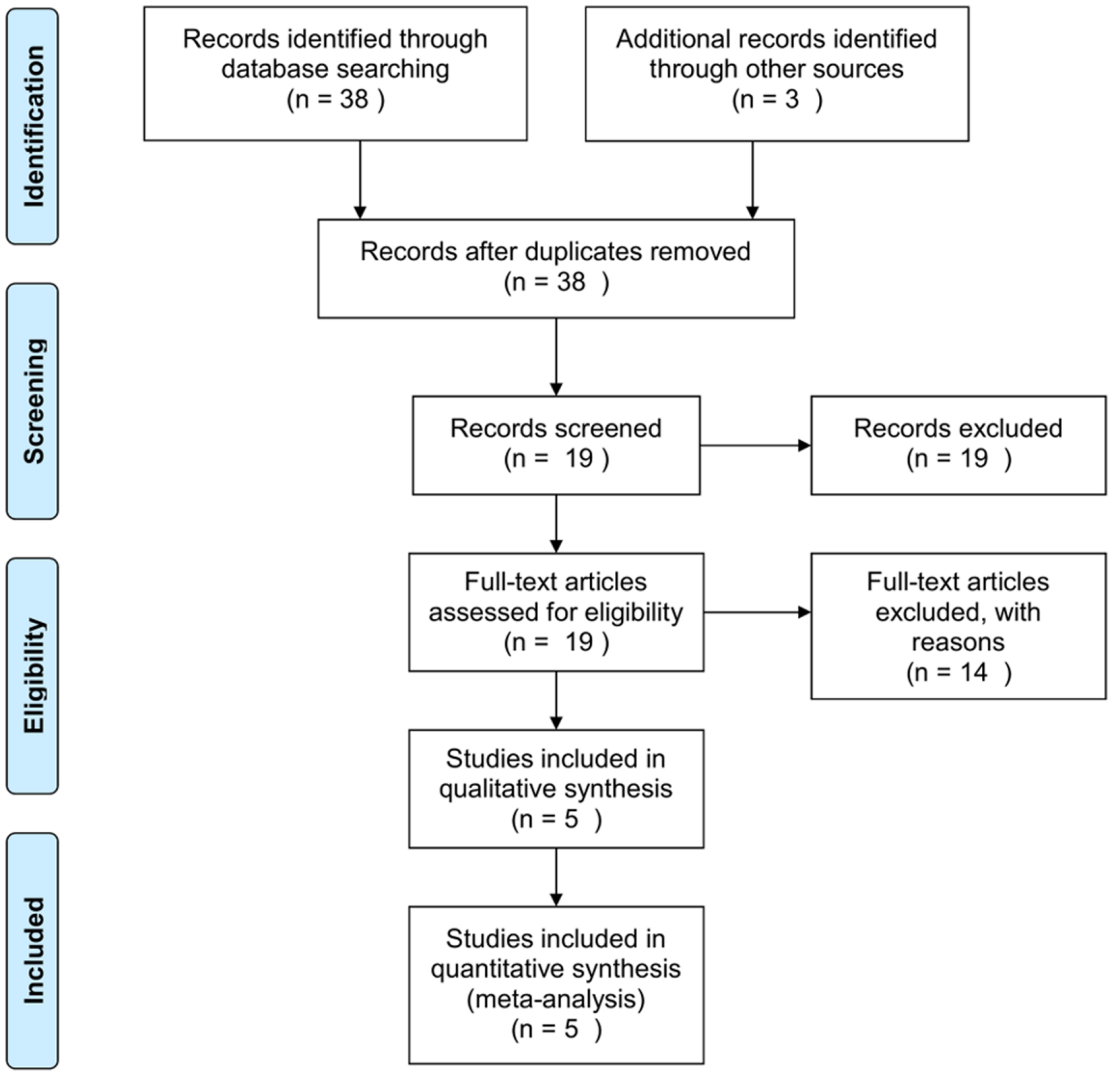
Data selection process. Search in the public microarray data repositories identified 38 microarray datasets involving cognitive impairments. We excluded 19 datasets that were either not relevant to our study or were not associated with any publication. We excluded 14 more studies as they involved different learning paradigms, test conditions, and outcomes in mice. We finally selected five studies that dealt with hippocampus dependent age-associated spatial learning in rats.

In order to reduce heterogeneity among studies selected for this meta-analysis we followed a conservative data selection process. We focused on datasets generated from carefully designed behavioural studies involving hippocampus dependent ASLI in Fischer 344 strain of male rats (*Rattus norvegicus*) as assessed by the Morris Water Maze (MWM). We mainly used the GEO (http://www.ncbi.nlm.nih.gov/geo/) and the ArrayExpress (http://www.ebi.ac.uk/array express/) microarray data repositories to search for microarray gene expression datasets using the keyword “memory and brain”. We also used the PubMed literature database to search for relevant studies (Figure 1). Affymetrix raw data (CEL files) for the selected studies were either directly downloaded from the GEO website or obtained through personal communication with the original authors.

### Data Preprocessing

All arrays were first assessed for image quality using the dChip software [17] (http://biosun1.harvard.edu/complab/dchip/). Minor contaminations present in a few of the arrays were corrected using the built in image gradient correction algorithm in dChip. The data quality was assessed using the RNA degradation ratios, relative log expression (RLE), and normalized unscaled standard errors (NUSE) plots using the *simpleaffy* and *affyPLM* packages in Bioconductor (http://www.bioconductor.org/) following standard procedures [18]. Arrays with bad quality e.g. variable background brightness, uneven hybridization, etc., or arrays having greater than 15% array outlier values were excluded. Within-study normalization and expression measurement were performed using the RMA methods [19] with default options in the *affy* package in R [20]. Within-study batch correction was performed using the Empirical Bayes method also known as the ComBat [21], which has been shown to produce better results than other comparable methods [22], [23]. Array hybridization dates were retrieved from CEL files and used as processing batches to perform batch correction. Age and spatial learning impairment were used as covariates.

### Data Integration

**Figure 2:**
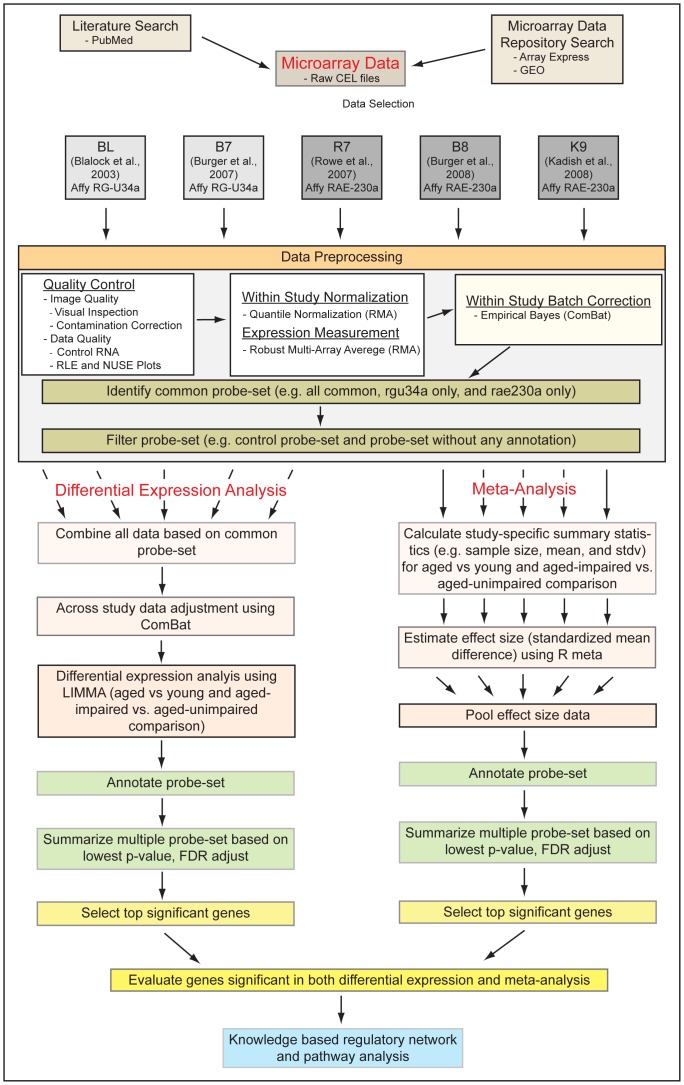
A summary of the meta-analysis workflow. Five individual studies (BL, B7, R7, B8, and K9) were selected for this meta-analysis. The studies involved two different array platforms, Affymetrix RG-U34a and RAE-230a. Following preprocessing, data were integrated across studies and across array platforms and analyzed in two ways: meta-analysis using random effect size model and differential expression analysis using the *limma* software. Top significant genes were used to identify enriched functions and pathways and to construct knowledge based gene regulatory networks using the Ingenuity Pathway Analysis software.

A common probe-set file that contains best matching pairs of probe-sets representing the same gene in the two chip types i.e. rgu34a and rae230a was downloaded from the Affymetrix website (www.affymetrix.com). Applying the common file, probe-sets from all studies belonging to the two different chip types were merged into three categories as follows: i) rg_exclu, probe-sets exclusive to the rgu34a chip type, ii) all5_com, probe-sets common among all five studies, and iii) rae_exclu, probe-sets exclusive to the rae230a chip type. Each probe-set specific data and their analysis outcome from all studies were combined in two ways (Figure 2): a) effect size integration, which combined the estimated effect size results and b) direct data integration, which combined the preprocessed data first before any analysis.

#### a) Effect size (ES) integration

Meta-analysis, which combines the results of independent but related studies in a relatively inexpensive way, has the ability to increase the statistical power to obtain a more precise estimate of gene expression differences. Though there are many ways to combine the results across studies, platform, and species [23], [24], [25], combining effect sizes using an inverse-variance method [26], [27] is considered to be the most comprehensive approach for meta-analysis of gene expression microarrays [24]. Therefore, we estimated effect sizes on the within-study batch-corrected data using the random effect size (ES) model as follows. First, for each probe-set, study-specific sample sizes, mean expression measures, and standard deviations were computed for each comparison. In order to understand the effect of age and spatial learning impairment, data were analyzed in two ways, e.g. by comparing samples across age (aged vs. young, AY) and across learning impairment (aged-impaired vs. aged-unimpaired, IU), respectively. Next, the *meta* package in R (http://cran.r-project.org/web/packages/meta/meta. pdf) was used to calculate each study-specific standardized mean difference (SMD) (Cohen’s d) for each probe-set, and later, probe-set SMDs for all studies in each category (e.g. rg_exclu, all5_com, and rae_exclu) were pooled utilising Hedges’ adjusted g [16] to obtain the final random ES for each probe-set. Effect size values for all probe-sets from all three categories were then combined together, annotated, and summarized. Duplicate probe-sets and multiple probe-sets annotated to the same gene were summarized by keeping the probe-set with the lowest p-value (of the z-value) for the gene [28]. Uninformative probe-sets were filtered out by removing probe-sets whose expression values had a coefficient of variation of zero across all arrays and probe-sets with a p-value (of the ES z-value) greater than 0.1. The p-values of the treatment effect for all probe-sets were adjusted for Benjamini and Hochberg (BH) multiple testing correction [29] in R.

#### b) Direct data integration

This was done by a cross-study and cross-platform normalization process by first combining data separately for each category (e.g. rg_exclu, all5_com, and rae_exclu) and then adjusting data across all studies. For each category, data were adjusted similarly as within-study batch correction, however, considering individual studies as separate batches. Next, differential expression (DE) analysis was performed by comparing the data in two ways as above e.g. AY and IU using the *limma* software package [30]. Significantly differentially expressed genes from all three categories were combined together, annotated, and summarized as described above. Duplicate and multiple probe-sets issues and multiple testing corrections were also handled similar to the ES analysis.

### Functional and Pathway Analysis

Functional and pathway analysis was performed mainly using the Ingenuity Pathway Analysis (IPA) software (http://www.ingenuity.com). Data sets containing identifiers of significant (p-value ≤0.05) differentially expressed genes from AY or IU comparisons with their corresponding ES estimates (as fold-change values) and p-values were used as input. Identifiers that were successfully mapped to their corresponding objects in the IPA knowledge base were considered for functional, network, and canonical pathway analysis.

For functional analysis the mapped identifiers that were associated with biological functions and/or diseases in the IPA Knowledge Base were considered. Right-tailed Fisher’s exact test was used to calculate a p-value determining the probability that each biological function and/or disease assigned to the data set is due to chance alone. The expression levels (up- or down-regulation) for all of the input genes in each function annotation category were compared with the information stored for those genes in the IPA Knowledge Base to predict whether the expression patterns correspond to the activation state (decreased or increased) for that category.

For network analysis the mapped identifiers were overlaid onto a global molecular network developed from information contained in the IPA Knowledge Base. Networks of network eligible molecules were then algorithmically generated based on their connectivity. Next, the functional analysis of a network identified the biological functions and/or diseases that were most significant to the molecules in the network based on the association of the network molecules with the biological functions and/or diseases in the Ingenuity Knowledge Base. Right-tailed Fisher’s exact test was used to calculate a p-value determining the probability that each biological function and/or disease assigned to that network is due to chance alone.

Canonical pathways analysis identified the pathways from the IPA library of canonical pathways that were most significant to the gene lists. All the mapped identifiers from the data set that were associated with a canonical pathway in the Ingenuity Knowledge Base were considered for the analysis. The significance of the association between the data set and the canonical pathway was measured in two ways: a) a ratio of the number of molecules from the data set that map to the pathway divided by the total number of molecules that map to the canonical pathway is displayed. b) Fisher’s exact test was used to calculate a p-value determining the probability that the association between the genes in the dataset and the canonical pathway is explained by chance alone.

## Results

### Data Selection, Preprocessing, and Integration

Search in the public microarray data repositories reveals that there is a large body of microarray data available involving cognitive impairments (Figure 1). Review of the resulting articles reveals that the goals of these studies are varied and include different learning paradigms, test conditions, subjects, and tissue types. After careful examinations of these datasets and following suitable data selection guidelines (see method), we identified five individual studies consisting of a total of 287 arrays (one animal per assay), which used two different Affymetrix chip types, RG_U34a and RAE230a (Table 1). The data represented young and aged rats that were learning unimpaired and aged rats that were learning impaired from a set of results published during 2003 to 2009. The selected datasets referred to as BL [31], B7 [4], R7 [32], B8 [15], and K9 [33] in this study allowed us to assess a combined gene expression changes related to aging, as well as ASLI in rats across multiple studies. These studies investigated spatial learning tasks in young (3–6 months old) and aged (24–26 months old) animals using the MWM as the training and assessment protocol. The BL and K9 studies were similar in design where only the unimpaired young and impaired aged animals were considered for comparison. The B7, R7, and B8 studies were similar in design where both young and aged groups had impaired and unimpaired animals as well as additional controls, e.g. cage controls, stress controls, and controls for visual impairment. A total of 265 arrays were finally selected following quality assessment (see methods, data preprocessing).

**Table 1:** Age-associated spatial learning impairment (ASLI) datasets for rats.

Dataset ID	Reference	Affymetrix Array Type	Number of Assays (one animal/array)
BL	Blalock et al. 2003 [31]	RG_U34	29
B7	Burger et. al. 2007 [4]	RG_U34	79
R7	Rowe et. al. 2007 [32]	RAE230a	50
B8	Burger et. al. 2008 [15]	RAE230a	80
K9	Kadish et al. 2009 [33]	RAE230a	49

**Figure 3:**
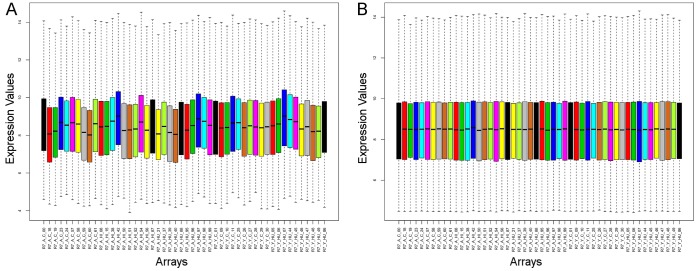
Boxplot of R7 dataset before (A) and after (B) RMA normalization. Each color represents a batch of arrays that were hybridized and processed at the same time.

**Figure 4:**
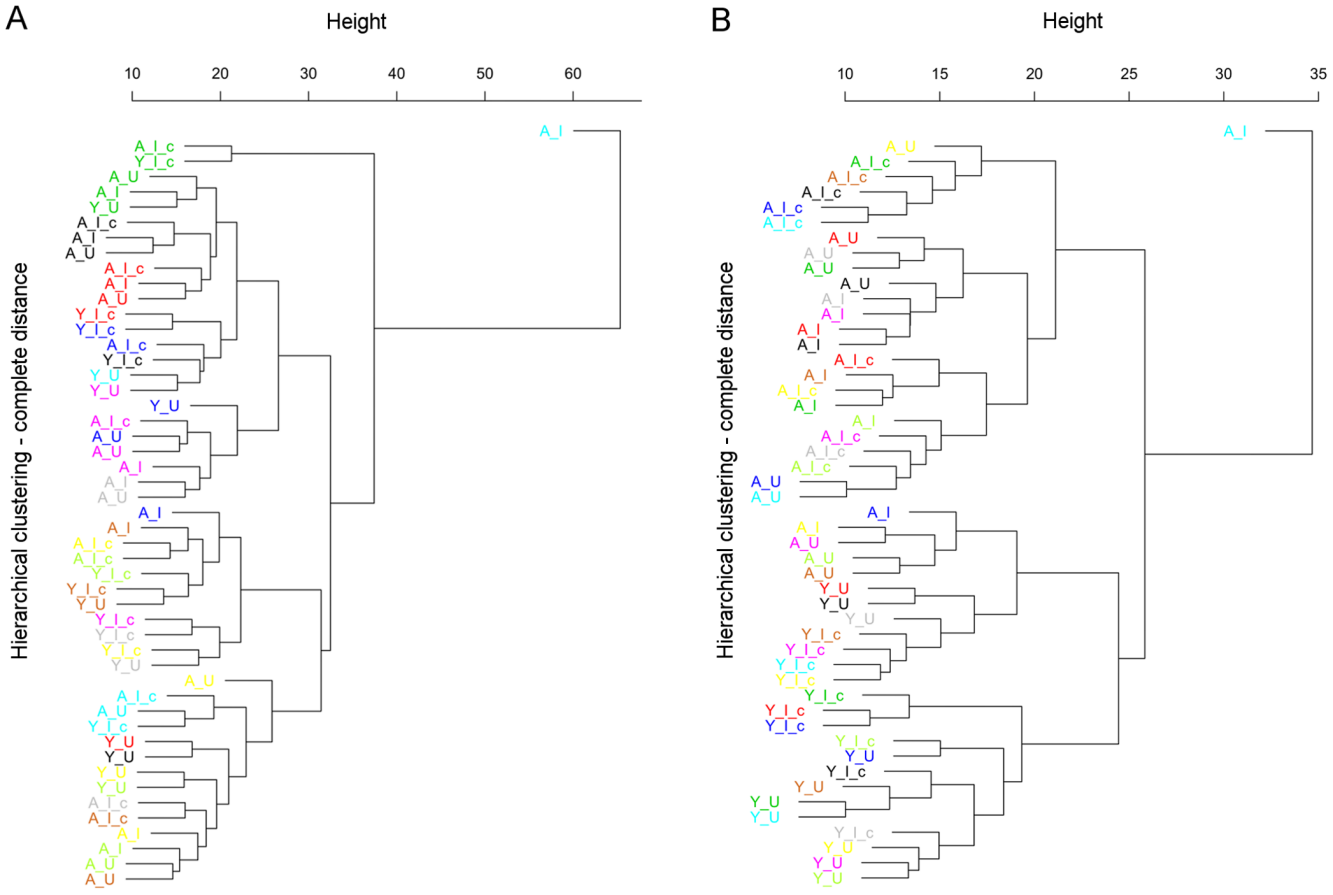
Hierarchical clustering of RMA normalized R7 data. Each color represents a batch of arrays, which were hybridized and processed at the same time. Batch effects are evident even after normalization and before batch adjustment (A) as arrays are mostly clustered in batches. However, following Empirical Bayes adjustment arrays are clustered based on aged and young phenotypes irrespective of batches (B). Leaf labels: A, aged; Y, young; I, impaired; U, unimpaired; c, control.

Hierarchical clustering analysis with normalized data shows that batch effects are clearly evident in all studies even after normalization (see Figures 3 and 4 for some representative results). Arrays that were hybridized on the same date as a batch are clustered together in the dendrogram. We used an Empirical Bayes method implemented in ComBat to remove batch effects. Batch effects were completely removed from the BL, B7, and K9 data and considerably removed from the B7 and B8 data. Data were integrated between the rgu34a chip which had a total of 8799 probe-sets and rae230a chip that had a total of 15923 probe-sets. After data integration, the rg_exclu category contained 2356 probe-sets exclusive to the rgu34a array only. The all5_com category included 6384 rgu34a unique probe-sets mapping to 5435 rae230a unique probe-sets that are common among all five studies. Finally, the rae_exclu category contained 10,431 probe-sets exclusive to the rae230a array type.

### Gene Identification and Functional Analysis

#### a) Aged vs. young (AY)

**Figure 5:**
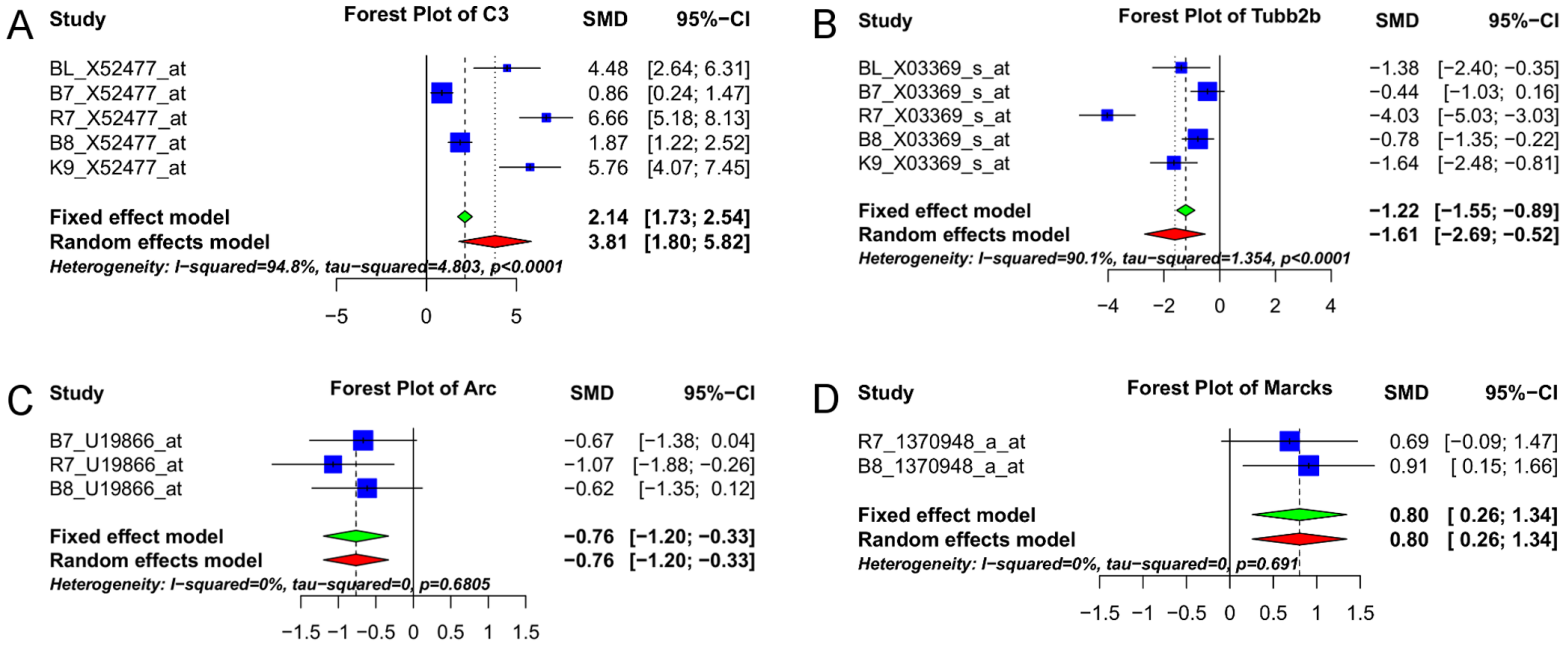
Forest plots of four representative significant genes. For the selected probe-set for each gene the individual study specific standardized mean differences (SMD) and their 95% confidence intervals (CI) are plotted and shown on each row. The effect size results are shown at the bottom of each plot. *C3* is up-regulated (A) and *Tubb2b* is down-regulated in the aged rats. *Arc* is down-regulated (C) and *Marcks* is up-regulated (D) in the aged-impaired rats.

In order to assess the effect of aging, a comparison was made between aged vs. young animals. After combining probe-sets from all three categories and after summarization we had ES estimates for 10,619 unique annotated genes. After filtering, there were 3235 genes left, of which 2245 genes were found significant with a p-value ≤0.05 (Table S1) and 1753 genes were found significant after BH multiple testing correction (pBH.ES ≤0.05). Among the 1753 genes, 874 genes have an I^2^ (ratio of true heterogeneity to total variation) value of 0% while 1347 genes have an I^2^ value under 40%. Table 2 shows the top 10 most up- and down-regulated genes in the aged animals compared to the young animals. The forest plots of two representative genes *C3* (complement component) (up-regulated) and *Tubb2b* (tubulin, beta 2B class IIb) (down-regulated) are presented in Figure 5A and B, respectively. DE analysis was performed on the data sets in parallel to the ES analysis. Using the 3235 genes from the ES analysis, the log fold-change (logFC) and p-values (pDE) for the corresponding differentially expressed genes were pulled out and BH adjusted similar to that of ES data. This resulted in a total of 1946 genes (pDE <0.05) and 1569 genes after BH adjustment (pBH.DE <0.05).

**Table 2:** Top ten most up- and down-regulated genes (based on ES) in the AY comparison.

Up-regulated genes							
Probe ID	Symbol	ES	z-value	p-value of z-value	pBH of z-value	LogFC of DE	pBH of DE
1398892_at	Npc2	3.988	3.16	0.002	0.009	0.474	0
X52477_at	C3*	3.812	3.716	0	0.002	0.730	0
X13044_g_at	Cd74*	3.389	3.148	0.002	0.009	0.916	0
M15562_g_at	HLA-DRA*	3.236	3.284	0.001	0.007	1.011	0
1368187_at	Gpnmb*	3.189	2.827	0.005	0.017	0.610	0
L03201_at	Ctss*	3.110	3.362	0.001	0.006	0.368	0
1373575_at	Fcer1g*	2.846	2.821	0.005	0.018	0.473	0
1370885_at	Ctsz	2.606	3.201	0.001	0.008	0.432	0
J03752_at	Mgst1*	2.544	3.229	0.001	0.024	0.362	0
1376652_at	C1qa*	2.519	3.709	0	0.007	0.488	0
**Down-regulated genes**							
**Probe ID**	**Symbol**	**ES**	**z-value**	**p-value of z-value**	**pBH of z-value**	**LogFC of DE**	**pBH of DE**
1376319_at	Sema3c*	−3.674	−3.867	0	0.001	−0.588	0
X57281_at	Glra2	−2.589	−4.599	0	0	−0.528	0
1388821_at	Trib2	−2.029	−2.578	0.01	0.028	−0.253	0
1388750_at	Tfrc*	−1.853	−2.576	0.01	0.028	−0.203	0
L03294_at	Lpl*	−1.803	−5.42	0	0	−0.395	0
1374966_at	Dcx*	−1.783	−3.42	0	0.005	−0.262	0
1389533_at	Fbln2	−1.756	−2.332	0.02	0.04	−0.192	0
D45412_s_at	Ptpro*	−1.721	−2.499	0.013	0.032	−0.319	0
M58369_at	Pnlip*	−1.618	−3.26	0.001	0.007	−0.200	0
X03369_s_at	Tubb2b*	−1.607	−2.907	0.004	0.015	−0.174	0

Top genes identified by IPA are indicated by an asterisk (*). Legends: ES, effect size; pBH, p-value with Benjamini and Hochberg correction; FC, fold change; DE, differentially expressed.

**Figure 6:**
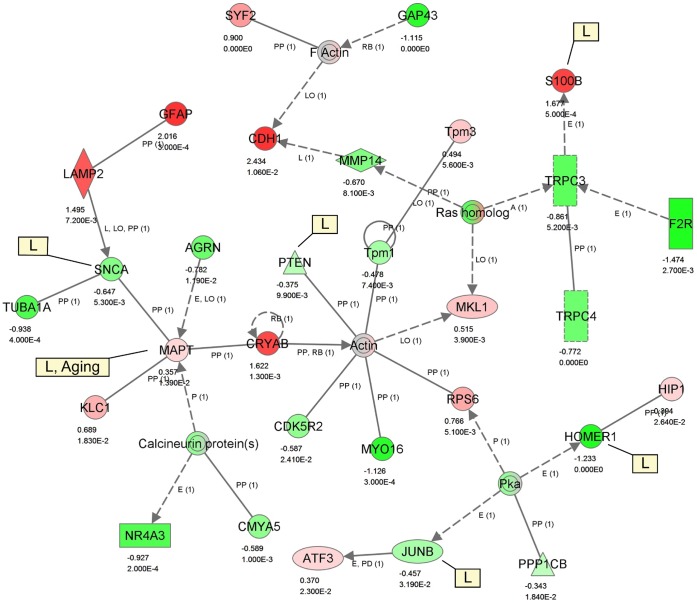
Network 3 from aged vs. young (AY) comparison. Major functions of this network are cellular assembly and organization, tissue development, and cell morphology. Each biological relationship (an edge) between two genes (nodes) is supported by at least one reference from the literature or curated information stored in the Ingenuity Knowledge Base. The intensity of the node color indicates the degree of up- (red) or down- (green) regulation observed in the AY comparison. The effect size and p-value for each gene is shown below the gene symbol. Edges are displayed with various labels that describe the nature of relationship between the genes (e.g. P for phosphorylation, PP for protein-protein binding, PD for protein-DNA binding, A for activation, E for expression, L for proteolysis, LO for localization, RB for regulation of binding). Any specific findings for a gene whether it is associated with aging (A), learning (L), and/or spatial learning (SL) is presented inside a rectangle beside that gene.

Functional and Pathway analysis were performed using the IPA software. For this analysis, we considered the significant genes based on unadjusted p-value (pES ≤0.05) of the random effect size, which resulted in a total of 2245 genes. These genes were used as input in the IPA of which 2240 were mapped to their corresponding object in the IPA Knowledge Base. The functional analysis identified the biological functions and/or diseases that were most significant (activation z-score value-cutoff of 1.980) to the mapped gene list. The IPA functional analysis predicts that comparatively more functions are decreased than increased in the aged animals. Table 3 shows a summary of the most significant functions, increased or decreased, as predicted by the IPA algorithm based on the expression levels of the genes in the dataset. The results show that the functions that are specifically decreased include cell viability of central nervous system cells, formation of cells, quantity and synthesis of inositol phosphate, and axonogenesis. Thus they affect the cell death and survival, cellular growth and proliferation, carbohydrate metabolism, molecular transport, small molecule biochemistry, cell morphology, and nervous system development and function in the aged animals. Major functions categories that see an increase are cellular movement, cellular development, and connective tissue development and function. The specific functions of the genes in this category include the migration of cells and differentiation of chondrocytes. We have generated biological knowledge based gene interaction networks for the AY significant genes. A representative network graph is presented in Figure 6, which shows the network interactions of some of the aging and learning genes. A summary of the functions for the top five most significant networks is given in Table 4. The most critical canonical pathways that are affected in the aged animals include Eif2 (eukaryotic translation initiation factor 2) signaling, antigen presentation, and Ox40 (tumor necrosis factor) signaling pathways (Table 5).

**Table 3:** Significantly increased or decreased functions in the AY comparison.

Functions Annotation	p-value	Predicted activation state	Activation z-score	High-level functions category	Genes
Cell viability of central nervous system cells	2.22E-03 to 2.35E-02	Decreased	−2.757 to −2.000	Cell death and survival	***ApoE*** **^A,L,SL^**, *Atf3*, *Bdnf* ^L,SL^, *Cdk5r1* ^L,SL^, *Cycs*, *Hspb1*, *Ide*, *Igf2*, *Ntf3* ^L^, *Plagl1*, *Prkcg* ^L,SL^, ***Rela*** **^L^,** *Serpini1*, *Sh3kbp1*, ***Slc11a2*** **^L^**, *Vegfa* ^L^, *Vip*
Formation of cells	7.96E-03	Decreased	−2.376	Cellular growth and proliferation	*Bdnf* ^L,SL^, *Egr1* ^L^, *Fgf18*, ***Icam1*** **,** *Igf2*, ***Nppa*** **, ** ***Pf4*** **, ** ***S100b*** **^L^**, *Sdc2*, *Wt1*
Quantity and synthesis of inositol phosphate	1.54E-02	Decreased	−2.186	Carbohydrate metabolism, molecular transport, small molecule biochemistry	*Agtr1*, *Avp* ^ L^, *Cckbr*, *Gal* ^L^, *Gnaq*, *Grp* ^L^, ***Icam1***, *Mas1*, *Pthlh*, *Rgs2*, ***Rgs3*** **,** *S1pr1*, *Trhr*
Axonogenesis	6.96E-03	Decreased	−1.980	Cell morphology, assembly and organization, nervous system development and function	***Actb*** **,** *Actr3*, *Agrn*, *Bdnf* ^L,SL^, *Cck*, ***Cntn2*** **^L^, ** ***Igf1r*** **,** *L1cam* ^L,SL^, ***Mbp*** **, ** ***Picalm*** **,** *Ppp2ca*, *Snap91*, *Stk11*
Migration of cells	8.08E-04 to 4.96E-03	Increased	2.158	Cellular movement	***Abcc1*** **,** *Actr3*, ***Agt*** **, ** ***Aif1*** **, ** ***Anxa2*** **, ** ***Bcar1*** **,** *Bdnf* ^L,SL^, ***C3*** **,** *Cck, * ***Ccl3l1*** **/** ***Ccl3l3*** **^L,SL^, ** ***Ccl5*** **, ** ***Cd44*** **, ** ***Cd82*** **,** *Cdc42*, **Dnm2**, *Drd5* ^L,SL^, *Gucy1a3*, *Gucy1b3*, ***Icam1*** **, ** ***Nfkbia*** **^L^,** *Ntf3* ^L^, *Pten* ^L^, *Reln*, ***Stat3*** **, ** ***Scpep1*** **,** *Tac1* ^L^, ***Tgfa*** **, ** ***Tgfa*** **, ** ***Tgfb1*** **, ** ***Tgfb2*** **, ** ***Tpm1*** **,** *Tubb2b*, *Vegfa* ^L^, and etc.
Differentiation of chondrocytes	1.54E-02	Increased	2.183	Cellular development, connective tissue development and function	***Grn*** **, ** ***Por*** **, ** ***Rela*** **^L^, ** ***Tgfb1*** **, ** ***Thrb*** **^L^**

Genes in bold were up-regulated and not bold were down-regulated in this analysis. Genes annotated as aging, learning, and spatial learning in the IPA knowledge base are indicated by “^A^”, “^L^”, and “^SL^”, respectively.

**Table 4:** Major functions associated with the top five networks in the AY comparison.

Network ID	Top functions associated with the networks	IPA score	Total focus genes
1	Molecular transport, cell-to-cell signaling and interaction, nervous system development and function	25	35
2	Endocrine system disorders, gastrointestinal disease, metabolic disease	21	33
3	Cellular assembly and organization, tissue development, cell morphology	17	30
4	Cell-to-cell signaling and interaction, cell signaling, molecular transport	14	28
5	Drug metabolism, protein synthesis, cancer	14	28

**Table 5:** Top canonical pathways in the AY comparison.

Name	p-value	Ratio
EIF2 signaling pathway	2.36E-07	58/170 (0.341)
Antigen presentation pathway	6.01E-05	14/27 (0.519)
OX40 signaling pathway	1.91E-04	19/60 (0.317)
Chondroitin sulfate degradation pathway	4.96E-03	6/14 (0.429)
IL-17A signaling in gastric cells pathway	5.17E-03	10/24 (0.417)
Complement system pathway	1.69E-02	10/32 (0.312)

#### b) Aged-impaired vs. aged-unimpaired (IU)

In order to assess the effect of ASLI, a comparison was made between the aged-impaired vs. aged-unimpaired (IU) rats where we included three sets of controls (e.g. cage controls, visual controls, and stress controls (no platform during memory test in the water maze)) in the aged-impaired group as was done in the B7 and B8 studies [4], [15]. After combining probe-sets from all three categories and after summarization there were 10,412 unique annotated genes with ES estimates. After filtering out uninformative genes there were 1310 genes left, of which 787 genes were found significant with a p-value ≤0.05 (Table S2). Among the 787 genes, 59 were significant with adjusted pBH.ES ≤0.05 and 55 of these genes have an I^2^ value of 0%. Table 6 shows the top 10 most up- and down-regulated genes in the aged-impaired as compared to the aged-unimpaired animals. Figure 5C and D show the forest plots of two representative genes *Arc* (activity-regulated cytoskeleton-associated protein) (down-regulated) and *Marcks* (myristoylated alanine-rich protein kinase C substrate) (up-regulated). DE analysis for the 1310 IU genes identified 460 significant genes (pDE ≤0.05), of which 92 were significant with pBH.DE ≤0.05. However, among the 92 genes significant in the DE analysis, 44 were also present in the ES meta-analysis (pES ≤0.05) category and 14 in the pBH.ES ≤0.05 category.

**Table 6:** Top ten most up- and down-regulated genes (based on ES) in the IU comparison.

Up-regulated genes								
Probe ID	Symbol	ES	z-value	p-value of z-value	pBH of z-value	LogFC of DE	p-value of DE	pBH of DE
1369775_at	Nucks1	1.187	4.105	0	0	0.129	0.008	0.074
S74393_s_at	Pax6	0.881	3.944	0	0.016	0.073	0.014	0.086
M27905_at	Rpl21	0.884	3.965	0	0.016	0.093	0.020	0.1
1388783_at	Hmgb1*	1.124	3.921	0	0.016	0.095	0.055	0.155
U93692_at	Nup88	0.814	3.665	0	0.026	0.083	0.004	0.056
J01436cds_s_at	CYTB	0.827	3.726	0	0.026	0.052	0.119	0.232
1373952_at	Prkag2	1.023	3.610	0	0.033	0.089	0.013	0.084
U78090_s_at	Alg10	0.780	3.522	0	0.033	0.061	0.041	0.135
AB002111_at	Pex12	0.780	3.534	0	0.033	0.100	0.001	0.034
1389373_at	Smad1*	0.949	3.375	0	0.04	0.099	0.045	0.144
**Down-regulated genes**								
**Probe ID**	**Symbol**	**ES**	**z-value**	**p-value of z-value**	**pBH of z-value**	**LogFC of DE**	**p-value of DE**	**pBH of DE**
1390518_at	Emid1	−1.259	−4.314	0	0	−0.063	0.049	0.148
rc_AA891838_at	Mrto4	−0.951	−4.224	0	0	−0.095	0.000	0.013
1389264_at	Ankrd54	−1.149	−3.983	0	0.016	−0.088	0.004	0.056
1369203_at	Wif1*	−0.980	−3.478	0	0.034	−0.056	0.023	0.107
U19866_at	Arc	−0.764	−3.46	0	0.034	−0.215	0.000	0.008
1376569_at	Klf2*	−0.960	−3.405	0	0.04	−0.182	0.000	0.013
rc_AA800613_at	Zfp36	−0.750	−3.396	0	0.04	−0.089	0.008	0.073
1398380_at	Vwa1	−0.94	−3.350	0	0.04	−0.098	0.002	0.038
1368451_at	Hrh3*	−0.940	−3.349	0	0.04	−0.094	0.005	0.063
S49760_g_at	Dgka	−0.711	−3.226	0.0013	0.051	−0.081	0.006	0.063

Top genes identified by IPA are indicated by an asterisk (*). Legends: ES, effect size; pBH, p-value with Benjamini and Hochberg correction; FC, fold change; DE, differentially expressed.

A total of 738 genes with significant effect sizes (pES ≤0.05) were used as input for the IU functional analysis in the IPA. Though cell viability of hippocampal neurons and CNS cells, cell-to-cell signaling, and molecular transport were the top functions in the results, none were statistically significant. However, when we reanalyzed with an effect size data set that was generated comparing the expression level of the aged-impaired animals with that of the aged-unimpaired animals without any controls, four functions e.g. molecular transport, cellular development, cellular growth and proliferation, and connective tissue development and function were significantly decreased (results not shown). The specific functions of these genes in these categories include transport of molecules and proliferation of fibroblast cell lines. In addition, growth of neuritis was also decreased among others. Similar to AY, we have generated biological knowledge based gene interaction networks for the IU related genes. A summary of the functions for the top five most significant networks is given in Table 7. The canonical pathways that are most affected in the aged-impaired compared to the aged-unimpaired animals include Nurr77 (nuclear receptor subfamily) signaling in lymphocytes, nNOS (nitric oxide) signaling in neurons, and glutamate receptor signaling (Table 8).

**Table 7:** Major functions associated with the top five networks in the IU comparison.

Network ID	Top functions associated with the networks	IPA score	Total focus genes
1	Neurological disease, tissue morphology	29	27
2	Cellular growth and proliferation, cancer, cell death and survival	16	19
3	Cell-to-cell signaling and interaction, nervous system development and function, carbohydrate metabolism	14	18
4	Cell death and survival, cellular development, hematological system development and function	10	15
5	Cell death and survival, metabolic disease, cellular function and maintenance	8	13

**Table 8:** Top canonical pathways in the IU comparison.

Name	p-value	Ratio
Nur77 signaling in T lymphocytes	6.13E-04	13/51 (0.255)
nNOS signaling in neurons	5.13E-03	12/46 (0.261)
Glutamate receptor signaling	5.68E-03	13/60 (0.217)
Calcium-induced T lymphocyte apoptosis	1.07E-02	12/57 (0.211)
Glutamate dependent acid resistance	1.48E-02	2/2 (1)

### Aging and Learning Related Genes

We searched the IPA Knowledge Base for genes that are annotated as aging related and genes that are annotated as learning related, particularly spatial learning. We found a total of 61 genes related to general aging, of which five, *Adraid* (all-trans retinoic acid-induced differentiation factor), *Aldoc* (aldolase C, fructose-bisphosphate), *Clu* (clusterin), *ApoE* (apolipoprotein E), and *Mapt* (microtubule-associated protein tau) (Figure 6) were present in our data set (p.ES ≤0.05). Further, there were 401 genes annotated as learning genes in the IPA Knowledge Base, of which 177 were categorized under spatial learning (SL). Among these learning genes 86 (30 of which were SL related) were present in our AY comparison (Table S3) and 48 (15 of which were SL related) were present in the IU comparison (Table S4) with p.ES ≤0.05. They were considered as the ASLI associated genes. Among the 86 genes for AY and 48 genes for IU, 15 were found common in both comparisons.

## Discussion

### Effective Meta-analysis Necessitates Proper Data Preprocessing and Integration

Meta-analysis has emerged as an essential tool in modern genetic and genomic analysis [34]. It can uncover a significant effect from a combined analysis as integration of a broader and/or richer collection of data has the potential to generate results that have greater confidence, and place less reliance on a single dataset [24], [34]. Although meta-analysis often includes large number of unrelated studies, we followed a more conservative approach in order to concentrate on microarray gene expression datasets that focused on the hippocampus dependent ASLI as assessed by MWM test. We started the data preprocessing with raw expression data (CEL files), which gave us the opportunity to perform consistent quality assessment, preprocessing, and filtering of imperfect arrays and outlier values. It also allowed correction of batch effects and removal of any unexplained technical variations. Our results (Figure 3 and 4) confirmed the findings of recent studies [21], [35] and demonstrated the necessity of removing batch effects from microarray data before integrating them in any analysis.

Next, we performed the random effect size meta-analysis by keeping the individual studies separate and then only combining the probe-set specific effects. We also performed the traditional differential expression analysis in parallel to the ES analysis after merging all probe-set data into a single pool through the process of cross-study and cross-platform data normalization (Figure 2). Even though the DE analysis was able to detect significant DE level, the difference was smaller compared to the ES. Overall, the ES analysis seems to be a better approach than DE analysis, particularly when combining data from different studies and platforms. Nonetheless, the DE results helped us verify the ES outcomes and better screen the aging and ASLI associated genes.

It is important to point out that during the data integration process we worked at the probe-set level rather than at the gene level. This is essential when combining data from independent microarray results from different platforms. Therefore, we integrated all data first before doing any filtering, annotation, and summarization. In the final filtering process we removed genes with a p-value (of the ES z-value) >0.1. Our observation is that, a gene may have a higher ES but not necessarily a lower p-value (Table S1 and S2). This is due to either the heterogeneity among studies or the fact that some datasets are lacking the expression information for that particular probe set. Also the genes whose treatment effect sizes are either zero or close to zero have higher p-values. These genes were therefore filtered out. Our data integration method has prevented any loss of information and generated a number of differentially expressed genes even after multiple testing corrections (Table S1 and S2) particularly for the AY comparison. It is also important to mention that ES estimates of some of these genes e.g. *C3* and *Tubb2b* (Figure 5, A and B) present some degree of heterogeneity. It is not unexpected in a meta-analysis as the heterogeneity may arise, as in this case, from differences in the details of MWM training, memory test and sample collection procedure, and other experimental variables pertaining to the individual studies. However, during the selection of the aging and ASLI related genes that had high heterogeneity, we made sure that the estimates of the ES are on the same direction.

In order to include more genes in the functional and pathway analysis using IPA we considered the unadjusted p-value (p≤0.05) of the random effect size for gene selection. Also, we analyzed data with lower number of genes following more stringent criteria such as using pES ≤0.005 (e.g. 888 genes) or pBH.ES ≤0.05 (e.g. 1753 genes) for AY comparison. It was satisfactory to note that the IPA analysis returned similar results. Also the expression levels (up- or down-regulation) identified in this meta-analysis for all or most of the genes in each function annotation category in the AY comparison correspond to the predicted activation state (decreased or increased) for that category as supported by the literature in the IPA Knowledge Base. Further, we were able to verify the results by literature review using the PubMed. The results support the fact that the genes and pathways identified in this analysis follow biological expectations. The genes identified (see below) are known to partake in aging and in learning impairments. This conclusion is also supported by follow up analysis including regulatory interaction networks based on known functions and interaction. The results obtained are discussed below in the context of aging and learning impairments associated with aging.

### Effect of Differential Gene Expression on Aging and Learning

Our IPA analysis has revealed major functions and pathways that are affected in the aged and aged-impaired animals. The results show that aging is affected by the genes functioning in cell viability, axonogenesis, and inositol phosphase metabolism. Further, these genes contribute to the imbalance in many major function categories including molecular transport, cell to cell signaling and interaction, and nervous system function. Considering the effect of the most significant differentially expressed genes on cellular biology, these genes could be classified into three distinct but non-exclusive categories: general aging (GA) genes that are associated with aging related disorders and not associated with any learning impairments, general aging genes associated with syndromic learning impairments (GASI), and general aging genes associated with non-syndromic learning impairments (GANSI). Given the confounding effect of aging on learning impairments one may expect an overlap in the three groups of genes. Below we summarize some key findings about some of the genes from each of the above three categories. These genes presented significant up- or down regulation in the AY and IU comparison (Table 2 and 6) and some of them were also identified as contributing to significantly increased or decreased function in the aged animals (Table 3).

#### GA or general aging genes

A majority of the genes that fall into this category were up-regulated in the aged in comparison to the young rats in our analysis and many have been implicated in disease vulnerability at old age in humans and animals. These GA genes may affect a number of pathways including Eif2 signaling, antigen presentation, complement system, and Ox40 signaling pathways (Table 5). EIF2 signaling is activated (through the phosphorylation of eIF2α) in response to a wide array of cellular stresses to protects cells by reducing the general rate of protein synthesis while facilitating programs of stress-induced gene expression [36]. OX40 is a member of the tumor necrosis factor (TNF) receptor family and plays a key role in the survival and homeostasis of effector and memory T cells and T-cell-mediated inflammatory diseases [37].

The GA genes that are of special interest to this discussion are *C3*, *Cd74* (CD74 molecule, major histocompatibility complex, class II invariant chain), *Ctss* (cathepsin S), *Ctsz* (cathepsins Z), *Agt* (angiotensinogen), *Mbp* (myelin basic protein), and *Cck* (Cholecystokinin). Specifically, *C3*, *Cd74*, and *Agt* expression level was increased (Table 3, migration of cells function) and they affect the endocrine system disorders, gastrointestinal disease, and metabolic disease functions. *C3* (Table 2 and Figure 5A) plays a central role in the activation of complement system and is needed to restore tissue injury. However, inappropriate or excessive activation of the complement system can lead to cell death and tissue destruction, thus contributing to further injury and impaired wound healing [38]. These consequences are clinically manifested in various disorders [39]. *Cd74* (Table 2) participates in several key processes of the immune system including antigen presentation, B-cell differentiation, and inflammatory signalling. Overexpression of *Cd74* has been reported in some inflammatory diseases and several forms of cancer (reviewed in [40]), and also known as an indicator of disease in some conditions. The longer form of CD74 also interacts with CTSS by direct binding [41], and both *Ctss* and *Ctsz* are also highly up-regulated in the aged rats (Table 2). Further, there is strong evidence implicating different AGT molecular variants as the cause of human essential hypertension and organ damage during aging (reviewed in [42]).

Expression of *Mbp* is known to decrease and *Cck* is known to affect the axonogenesis function in the aged animals (Table 3). Our analysis has revealed an increased expression of *Mbp* and a decreased expression of *Cck*. MBP is a major constituent of the myelin sheath of oligodendrocytes and has an important role in the pathophysiology of multiple sclerosis [43], which is a chronic inflammatory and neurodegenerative disease of the CNS of unknown cause. *Cck* is extensively expressed in the brain and a number of diverse changes to hippocampal *Cck* expression profile have been documented in various models of epilepsy [44]. *Cck* is also known to have a role in modulating the neuronal network of anxiety and panic disorder that involves other parts of the brain e.g. amygdale, hypothalamus [45]. Such results argue that the GA genes in general are associated with reduction in physiological and immunological efficiency leading to deterioration (senescence) with advancing age in the aged rats.

#### GASI or general aging genes associated with syndromic learning impairments

Deterioration of mental and physical state is very common with advancing age, which manifest in various syndromes. It is apparent that many syndromes associated with aging are also involved in memory loss and learning impairments. One such syndrome is the Alzheimer’s disease (AD), which has been studied extensively. Among the identified GASI genes in this analysis that have been implicated in AD or late-onset Alzheimer disease (LOAD) include *ApoE* [46], [47], *Mapt* [48], *Igf1r* (insulin-like growth factor 1 receptor) [49], *Clu* [50], [51], *Picalm* (phosphatidylinositol binding clathrin assembly protein) [50], [51], *Cdk5r1* **(**cyclin-dependent kinase 5, regulatory subunit 1, p35) [52], and *Ide* (Insulin degrading enzyme) [53]. *ApoE*, *Mapt*, *Igf1r*, *Clu*, and *Picalm* were up-regulated, and *Cdk5r1* and *Ide* were down-regulated in the aged animals compared to the young. These GASI genes may also lead to syndromic learning impairments by affecting various key neuronal functions. For example, *ApoE*, *Cdk5r1* and *Ide* are known to decrease cell viability and *Picalm* and *Igf1r* are known to affect axonogenesis (Table 3).

Specifically, *ApoE* and *Mapt* have been annotated as the aging and learning genes in the IPA Knowledge Base (Table S3). *ApoE* gene is known as the strongest risk factor for age-related cognitive decline during normal ageing [54]. APOE isoforms differentially regulate Aβ (amyloid β-peptide) aggregation and clearance in the brain, and have distinct functions in regulating brain lipid transport, glucose metabolism, neuronal signalling, neuroinflammation, and mitochondrial function (reviewed in [55]). Toxicity of Aβ also depends on *Mapt* (Figure 6). Increase in MAPT levels may represent a very early sign of NFT (neurofibrillary tangle) formation and AD in humans [48]. Down-regulated *Igf1r* activity has been implicated with prolonged human lifespan [49]. When considering age-related neurodegeneration in AD, signalling through the *Igf1r* is disturbed in the AD patients’ brain, and an increased level of *Igf1r* has been reported in the degenerating synapses in cerebral cortex within and surrounding Aβ plaques in people with AD compared to people of the same age without the disease (reviewed in [49]). Through the deregulated activity of *Cdk5*, *Cdk5r1* is involved in the pathology of AD [52], synaptic plasticity, learning, and memory [56]. IDE is involved in the degradation of Aβ and other bioactive peptides e.g. insulin and IGF-1 and IGF-2 in vitro (reviewed in [57]). PICALM plays a critical role in iron homeostasis and cell proliferation [58]. PICALM knockdown can result in reduced APP (amyloid precursor protein) internalization and Aβ generation, while overexpression can cause increased APP internalization and amyloid plaque load [59]. Irregularities in the Aβ clearance pathway are thought to initiate Aβ and tau protein accumulation in specific brain regions and consequent toxic events that lead to synaptic dysfunction and neurodegeneration in AD. This is associated with the progressive destruction of synaptic circuits controlling memory and higher mental function.

Besides the above genes associated with AD, there is a number of GASI genes associated with other age-related disease syndromes and related memory impairment. For example, *Cntn2* (Contactin-2), a learning gene, is up-regulated in the aged (Table S3), *Hmgb1* (high mobility group box-1) is up-regulated in the aged-impaired (Table 6), and *Tubb2b* is down-regulated in the aged rats (Table 2). *Cntn2* plays a role in the formation of axon connections [60] and autoimmune responses to *Cntn2* have been implicated in multiple sclerosis [61]. Studies show that cellular stress, trauma, and inflammatory condition can also result in the up-regulation of *Hmgb1* in the hippocampus in aged rats, which results in reduced cognitive function in a reversal learning version of the MWM test [62], [63]. Further, *Tubb2b* is a major component of microtubules cytoskeletal structures essential for cell motility and function and one of the top ten most down-regulated genes in the AY comparison (Table 2). A spectrum of neurological disorders (e.g. Polymicrogyria) characterized by abnormal neuronal migration, differentiation, organization, axon guidance, and maintenance has recently been associated with various mutations in *Tubb2b* [64], [65]. In summary, a number of genes identified in the aged and aged-impaired animals are associated with a number of syndromes and fall in the category of GASI genes, which may contribute to the memory loss and learning impairments observed in the aged-impaired animals.

#### GANSI or general aging related genes associated with non-syndromic learning impairments

It is apparent that the majority of the differentially expressed genes in the aged or aged-impaired animals are known to facilitate learning and memory formation and are not implicated in any syndromes. They have been annotated as learning or spatial learning genes in the IPA Knowledge Base (Table S3 and S4). The canonical pathways that are most relevant to the GANSI genes functioning in the brain include nNos signaling pathway and glutamate receptor signaling pathway, which were identified most significant in the IU comparison (Table 8). nNos [66], [67] and glutamate receptors (reviewed in [68]) play an important role in neurotransmission and are critical to LTP, memory formation and synaptic plasticity.

The genes that deserve particular attention in the GANSI category are the 59 genes identified in the IU comparison following BH correction on the effect sizes (pBH.ES ≤0.05). These genes were differentially expressed in the aged rats with spatial learning impairment as compared to those without spatial learning impairment. *Arc*, a learning gene, is one of the most interesting of these 59 genes and is among the top ten most down-regulated genes in the aged-impaired animals (Table 6). The immediate-early gene *Arc* (aka *Arg3*) (Figure 5C) expression is found vital for spatial memory consolidation and long-term synaptic plasticity in a variety of hippocampal-dependent and hippocampal-independent tasks, including spatial learning in the MWM (reviewed in [69], [70]). *Arc* is known for its tight experience-dependent regulation, dendritic mRNA transport, and local protein expression in activated synapses. For example, blocking *Arc* expression either using *Arc* knockout mice [71] or intra-hippocampal injections of *Arc* antisense oligonucleotides [72] is known to impair or prevent LTP without affecting short-term memory performance.

When we considered the larger list of 787 differentially expressed genes in the IU comparison (BH uncorrected, pES ≤0.05), we also found 48 genes annotated as learning or spatial learning genes in the IPA Knowledge Base (Table S4). Some of the interesting learning genes among theses 48 genes include *Camk2a* (calcium/calmodulin-dependent protein kinase II alpha), *Creb1* (cAMP responsive element binding protein 1), *Crem* (cAMP responsive element modulator), *Egr1* (early growth response 1), *Homer 1* (homer homolog 1) (Figure 6), *Junb* (jun B proto-oncogene) (Figure 6), *Psen2* (presenilin 2), *Slc11a2* (solute carrier family 11), and *Marcks*. Particularly, *Marcks* (pES = 0.004) (Figure 5D) is highly up-regulated in the aged-impaired animals. Timofeeva and colleagues recently reported that local infusions of MARCKS long peptide into the rat hippocampus resulted in a dramatic impairment of both working and reference memory in a dose-dependent manner with robust impairment at higher doses [73], most likely through the inhibition of alpha7 nicotinic acetylcholine receptors [74]. Thus, our analysis has identified the two genes, *Arc* and *Marcks*, as prime candidates for further investigation for their role in ASLI.

Additional GANSI genes include *Bdnf* (brain-derived neurotrophic factor), *Ntf3* (neurotrophin 3), *Igf2*, *Serpini1* (neuroserpin), *Gucy1a3* (guanylate cyclase 1, soluble, alpha 3), *Gucy1b3* (guanylate cyclase 1, soluble, beta 3), *Avp* (arginine vasopressin), *Gnaq* (guanine nucleotide binding protein), *Grp* (gastrin releasing peptite), *Pthlh* (parathyroid hormone-like hormone), *Trhr* (thyrotropin-releasing hormone receptor), *Agrn* (agrin), *L1cam* (Cell adhesion molecule L1), and *Ppp2ca* (protein phosphatase 2, catalytic subunit, alpha isozyme). These differentially expressed genes in the AY comparison play a critical role in the increase or decrease of several significant functions (Table 3) in the aged animals. Since a majority (73%) of the aged animals in the AY comparison were also impaired in the spatial learning task, it is not surprising that some of the aging genes may also contribute to the ASLI in these animals. Below we highlight some major functions of these GANSI genes.

For example, the genes *Bdnf*, *Ntf3*, *Igf2*, and *Serpini1* were down-regulated in the aged animals and are known to decrease cell viability of CNS cells (Table 3). Neurotrophins such as *Bdnf* and *Ntf3* expression is strongly associated with synaptic function and plasticity. Specifically, *Bdnf* is known as a strong mediator for LTP (long term potentiation) in the hippocampus and play an essential role in memory formation in the adult brain (see review [75]). *Igf2* is a late response gene regulated by the CREB-C/EBP pathway and plays a critical role in memory consolidation and enhancement [76]. Furthermore, injections of recombinant IGF-II into the hippocampus after either training or memory retrieval significantly enhance memory retention and prevent forgetting. Neuroserpin e.g. *Serpini1* expression is involved in regulating the proteolytic balance associated with axonogenesis and synaptogenesis during development and synaptic plasticity in the adult [77], [78].

Further, *Gucy1a3* and *Gucy1b3* are involved in the increase of cellular movement function (Table 3). They are soluble guanylate cyclises (sGC) and are part of the nitric oxide (NO)/sGC/cGMP dependent protein kinase (PKG) signaling pathway that plays a key role in memory processing [66], [67]. Inhibition of sGC, of PKG or of cGMP-degrading phosphodiesterase has been found to impair LTP [79]. Both GUCY1A3 and GUCY1B3 were found down-regulated in the aged animals, which may explain the ASLI in these animals.

The products of the genes *Avp* [80], *Gnaq* [81], *Grp* [82], *Pthlh* [83], and *Trhr* [84] maintain the quantity and synthesis of IP_3_ (inositol 1,4,5-triphosphate) level in the cell (Table 3) and are down-regulated in the aged rats. These genes facilitate IP_3_ production in the brain and some through the activation of phospolipase C (PLC) [80], [81], [82], [83], [84]. Some of them e.g. AVP [85] and GRPs (see review [82]) are specifically involved in regulating cognition and memory. IP_3_ is an important second messenger in the neuron produced from phosphatidylinsitol biphosphate (PIP_2_) and cleaved by PLC. IP_3_ binds to IP_3_ receptors, which are gated Ca^2+^ channels that release calcium from the endoplasmic reticulum in to the cytosol [86]. Ca^2+^ in turn controls many different signalling events within neuron, including neurotransmitter release and gene expression in the cell nucleus. At least two Ca^2+^-activated protein kinases e.g. Ca^2+^/calmodulin-dependent protein kinase (CaMKII) and protein kinase C (PKC) have been implicated in LTP induction. LTP is the underlying cellular molecular mechanism that correlates with learning and memory formation [87]. Thus, down regulation of the genes *Avp*, *Gnaq*, *Grp*, *Pthlh*, and *Trhr* can have a negative effect on the Inositol phospholipid-calcium-CamK-protein kinase C transduction pathway through decreased quantity and synthesis of IP_3_ in the aged animals and directly or indirectly contribute to age-associated non-syndromic learning impairments such as ASLI.

A number of genes e.g. *Agrn*, *L1cam*, *Ppp2ca* that are down-regulated in the aged animals demonstrated spatial learning impairment. Lower expression of these genes is known to decrease axonogenesis (Table 3). They play a critical role in neurite outgrowth, synaptogenesis, and synaptic plasticity. For example, high level of *Agrn* (Figure 6) expression was found in regions of the adult brain that show extensive synaptic plasticity. Recent studies demonstrated a substantial loss of excitatory synapses in the adult transgenic mice brain that lacked agrin expression. Further, they demonstrated inhibition of synaptogenesis by agrin antisense oligonucleotides or agrin siRNA in neuronal cell culture (reviewed in [88]). *L1cam* promotes the outgrowth of neurites and thereby contributes to formation of neuronal connections, learning, and memory [89], [90] via activation of the mitogen-activated protein kinase (MAPK) pathway [91]. *Ppp2ca* (aka Pp2a) is involved in Ca2+-dependent dephosphorylation of SNAP-25 [92] and SNAP-25 phosphorylation plays an important role in neural plasticity and long-term potentiation in the hippocampus [93].

It is important to note that Fischer 344 strain of rats have a median life-span of 23–31 months in captivity [94], [95]. Their normal age-related incidence of neoplasms and degenerative diseases is high, particularly, once the rats pass 24 months of age [94], [95]. Also, the effect of aging and ASLI on brain gene expression is evident in the aged (24–26 months old) in comparison to the young (3–6 months old) rats as discussed above. Indeed, it is expected that studies on animals beyond 26 weeks of age may show involvement of additional genes in this phenomenon and the effects observed could be more pronounced at later stages of the rat’s life-span.

In conclusion, we report that aged animals display a significant decrease in cell viability, axonogenesis, and inositol phosphate metabolism. They also show a significant increase in the migration of cells and differentiation of cells functions due to the altered gene expression. The regulatory interactions of the differentially expressed genes seems to affect molecular transport, cell to cell signaling and interaction, nervous system development and function, and cell death and survival. The genes that are known to be involved in the above functional changes and/or those present most significant expression changes in the aged or aged-impaired animals could be broadly classified into three major categories such as GA, GASI, and GANSI. The GA genes are mostly involved in inflicting various aging related senescence (e.g. stress, disorders, and inflammation conditions) and generally are not associated with any learning impairment. The GASI genes, on the other hand, are associated with age-related neurological disease syndromes e.g. Alzheimer’s disease, which generally affect normal cognitive functioning and may result into syndromic memory impairments. The most important group of genes perhaps is the GANSI genes, most of which show down-regulation in the aged or aged-impaired rats and by themselves usually are not associated with any syndromes. These genes affect various signal transduction pathways and functions in the brain contributing to the disruption of proper learning and memory formation. We suggest that the GANSI genes should form the foundation for future studies in understanding age-associated memory impairments such as ASLI. These GASI and GANSI genes form a set of interesting candidates for future investigations as to how they interact with each other, how they are regulated, and what target genes they may affect in order to elucidate the mechanisms behind aging and age-associated spatial learning impairment.

## Supporting Information

Table S1 **Details of all 2245 significant genes in the AY comparison.**
(XLSX)

Table S2 **Details of all 787 significant genes in the IU comparison.**
(XLSX)

Table S3 **List of 86 learning genes found in the AY significant genes.**
(XLSX)

Table S4 **List of 48 learning genes found in the IU significant genes.**
(XLSX)

Checklist S1 **Preferred reporting items for systematic reviews and meta-analyses (PRISMA) checklist.**
(DOCX)
